# When Does the Timing of an Urgent Endoscopy for Foreign Body Extraction Matter?

**DOI:** 10.7759/cureus.25143

**Published:** 2022-05-19

**Authors:** Yara Dababneh, Patrick Brown, Suraj Suresh, Eva Alsheik, Jason Schairer

**Affiliations:** 1 Internal Medicine, Henry Ford Health System, Detroit, USA; 2 Division of Gastroenterology and Hepatology, Henry Ford Health System, Detroit, USA

**Keywords:** ingestion, magnets, timing, developmental delay, endoscopy, foreign body

## Abstract

Most cases of foreign body ingestion are managed by observation without the need for endoscopic intervention as these objects are expected to pass spontaneously. However, urgent endoscopy is indicated depending on the type and size of the ingested material to avoid the risk of perforation. We present a case of repeated foreign body ingestion in a patient with a developmental disability and illustrate the importance of patient selection for urgent endoscopy.

## Introduction

Foreign body ingestion and food bolus impaction occur relatively commonly; however, studies have shown that 80% or more of ingested foreign objects will likely pass without the need for intervention [1,2]. Foreign body ingestion in adults occurs most often in individuals with psychiatric disorders, developmental delays, and in those who are incarcerated [1]. Two recent studies have shown that the rate of endoscopic intervention (63% to 76%) in the setting of intentional foreign body ingestion is higher than the need for surgical intervention (12% to 16%) [3,4]. Guideline recommendations state that the timing for surgical or endoscopic interventions depends on the material ingested and its location without taking into consideration developmental disabilities and any imprecise communication, which have the potential to lead to adverse patient outcomes [5].

## Case presentation

A 58-year-old man with a developmental disability residing in a group home presented with abdominal pain, nausea, and vomiting for one day after a reported foreign body ingestion, as reported by his caregiver. The patient was hemodynamically stable with a benign abdominal examination. On a previous occasion, the patient presented with similar symptoms and was found to have a small bowel obstruction from the ingestion of multiple foreign bodies that required exploratory laparotomy. In the current presentation, an acute abdominal series revealed multiple ingested metallic foreign bodies. A subsequent computed tomography scan showed multiple metallic foreign bodies within the duodenum, terminal ileum, and colon. The presence of magnets was not suggested in either imaging study report. Indications for emergent endoscopy were not met based on the imaging results; however, given the patient’s history of foreign body ingestion leading to small bowel perforation, an emergent endoscopy was performed rather than monitoring for peristaltic passage with serial imaging. 

**Figure 1:**
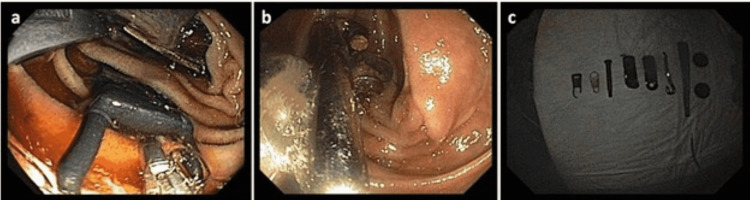
(a and b) Endoscopic images from the upper endoscopy showing several metallic objects within the duodenal lumen. (c) The objects removed include (left to right) two zippers, one unidentified plastic object, two brackets, one hook, one broken spoon, and four magnets.

The patient underwent esophagogastroduodenoscopy, resulting in the retrieval of two zippers, one unidentified plastic object, two brackets, one hook, one broken spoon, and four magnets from the duodenum (Figure 1). Object retrieval was performed with the aid of rat-toothed forceps, a 13-mm snare, and a Roth net. After complete retrieval of objects, the upper gastrointestinal tract up to the proximal jejunum was examined, and no remaining foreign bodies were seen. Serial abdominal X-rays showed foreign bodies in the colon with no signs of advancement through the colon. The patient subsequently underwent colonoscopy for the retrieval of a screw and a nail, among various other small metallic objects. Post-procedure imaging did not reveal any residual foreign bodies. The patient’s case was discussed with adult protective services to assess the safety of his living situation at the group home, and the patient was discharged in stable condition.

## Discussion

Initial management of foreign body ingestion focuses on airway management and the timing of endoscopic intervention. In patients who are unable to protect their airways or have the risk of aspiration, ventilator support is warranted [5]. Current guidelines delineate the need for and timing of endoscopic intervention based on the patient's age, characteristics of the object (size, shape, and anatomic location), and the time elapsed since ingestion [6,7]. According to the American Society for Gastrointestinal Endoscopy guidelines, patients who are clinically stable without overt symptoms that suggest gastrointestinal obstruction and/or the presence of esophageal foreign bodies or food impactions usually do not require urgent endoscopy within 24 hours [1,2].

In our patient’s case, standard practice guidelines would have recommended serial imaging because all foreign bodies were post-pyloric based on imaging [8,9]. However, given the patient’s prior history of foreign body ingestion requiring surgery and the potential for miscommunication due to his developmental disability, a decision was made to pursue urgent endoscopic retrieval. The procedure resulted in the successful removal of several objects, including multiple magnets. Magnet ingestion can cause severe gastrointestinal injury because the attractive force between magnets or with other metal objects can lead to entrapment of objects within a portion of the bowel wall, potentially causing subsequent bowel wall necrosis, fistula formation, perforation, obstruction, volvulus, or peritonitis [8]. Current guidelines advocate for earlier endoscopic intervention when ingestion of magnets is suspected [5]. In our case, neither the available imaging nor the history was suggestive of magnet ingestion and observation would have been the recommended approach. However, in hindsight, this might have led to the adverse effects described above.

## Conclusions

We advocate for early invasive strategies regardless of the location of the foreign object within the gastrointestinal tract when managing foreign body ingestion in patients with a history of prior ingestions and where the potential for imprecise communication arises, especially in cases in which there is a repeated history of foreign body ingestion related to developmental disability. This practice will help prevent adverse events and unnecessary surgery and reduce mortality.
